# Understanding Sociocultural Factors Shaping Married Men's Vasectomy Decisions: A Qualitative Study in Bihar

**DOI:** 10.7759/cureus.107709

**Published:** 2026-04-25

**Authors:** Ekta Krishna, Akash P Zanwar, Parvati Roy, Shamshad Ahmad, Pragya Kumar, Sanjay Pandey

**Affiliations:** 1 Community and Family Medicine, All India Institute of Medical Sciences, Patna, IND

**Keywords:** family planning, male sterilization, qualitative research, sociocultural barriers, vasectomy

## Abstract

Wider acceptance of vasectomy among married men can play an important role in controlling population growth in India. It helps take shared contraceptive responsibility and reduces the health risks and social burden that often fall on women. This study mainly aimed to explore the barriers and perceptions influencing vasectomy acceptance among married men and to identify strategies that could enhance its uptake as a family planning option. A descriptive qualitative study was conducted between March and October 2024 in urban and rural field practice areas of a tertiary care hospital in Bihar. Eighteen married men were recruited using purposive sampling, and in-depth interviews were conducted until thematic saturation was achieved. Data were analyzed using thematic analysis. Participants reported persistent myths and misconceptions about vasectomy, including fears of impotence, weakness, and infertility. Historical distrust stemming from coercive sterilization campaigns and entrenched gender norms placing family planning responsibility on women further discouraged uptake. Spousal disapproval, fear of irreversibility, and social stigma around masculinity were frequently cited. Suggested solutions included community-based education, peer advocacy by satisfied vasectomy users, and counseling by Accredited Social Health Activist (ASHA) workers and other frontline health professionals. Misinformation, gender norms, and historical mistrust are significant barriers to vasectomy acceptance in Bihar. Targeted interventions, including myth-busting campaigns, male-inclusive counseling, and strong government promotion, are needed to normalize vasectomy as a safe, acceptable contraceptive choice.

## Introduction

Vasectomy is a safe, simple, and effective method of family planning [1]. It can be performed in low-resource settings and may serve as a practical alternative to tubal ligation for women [2]. Despite being a simpler and safer procedure than tubectomy, vasectomy remains poorly accepted in India [3]. According to the National Family Health Survey-5 (NFHS-5), vasectomy accounts for <1% of contraceptive use in India, at around 0.3%, despite the availability of a cash incentive of ₹3000 [4].

Although men often occupy a dominant role in household decision-making, the responsibility for family planning continues to fall disproportionately on women in many settings in India. At the same time, India has one of the largest population burdens globally, underscoring the importance of strengthening effective and acceptable family planning strategies [5]. Temporary contraceptive methods, while useful, may be limited by issues such as repeated use, social stigma, inconvenience, and lower long-term acceptability in some contexts. In contrast, permanent methods offer a one-time solution; however, female sterilization continues to be far more common than male sterilization in India [6].

Previous studies suggest that poor uptake of vasectomy is influenced by limited awareness, misinformation, and sociocultural beliefs surrounding the procedure [7]. Common misconceptions include fears of impotence, weakness, infertility, loss of libido, or impaired sexual performance. Cultural and religious beliefs, along with stigma related to masculinity and male contraceptive responsibility, may further discourage men from considering vasectomy as a family planning option. These barriers indicate that nonacceptance of vasectomy is shaped not only by lack of information but also by broader social and gender norms.

Although studies from India have examined awareness and attitudes toward vasectomy, there is limited context-specific evidence from Bihar exploring married men’s perceptions and the sociocultural factors influencing its acceptability. Understanding these issues in Bihar is particularly important because contraceptive decision-making is often shaped by local beliefs, gender relations, family dynamics, and community norms. Therefore, this qualitative study was conducted in the field practice areas of a tertiary care hospital in Bihar to explore married men’s awareness, perceptions, and sociocultural barriers related to vasectomy acceptance and to identify suggestions that may help improve its acceptability as a family planning method.

## Materials and methods

Study design and setting

This study employed a descriptive qualitative design to explore the factors influencing the acceptance of vasectomy among married men. The study was conducted at urban and rural training centers under the field practice area of All India Institute of Medical Sciences (AIIMS), Patna. Data collection took place between March 21, 2024, and October 21, 2024.

Study participants

Participants were recruited using purposive sampling. Eligible participants were married men aged 18 years or older attending outpatient departments (OPDs) at the study sites. At the rural center, individuals were approached and given a brief explanation of the study’s purpose and objectives. Because vasectomy is a sensitive and stigmatized topic in the local context, individuals who were willing, comfortable, and able to engage in an in-depth discussion were invited to participate, as they were more likely to provide rich, relevant information. Those who did not provide consent were excluded from the study. At the urban center, with the assistance of Mr. XYZ, participants were directed one by one to a private and quiet room for the interviews. Recruitment was conducted face-to-face by the research team, and a total of 18 participants consented to participate in the study. Interviews were conducted between 9 am and 5 pm, i.e., the working hours at the respective centers.

Research team

The study was led by PK and SA, senior faculty members in the Department of Community and Family Medicine at AIIMS, Patna, both experienced in qualitative research. The team also included EK, APZ, and PR, who were trained in qualitative data collection and analysis methods. All researchers were fluent in Hindi (the local language), ensuring effective communication and data processing. Team members disclosed their professional background and role in the study to participants, maintaining reflexivity throughout the research process.

Study tool

A semi-structured open-ended interview guide was developed by PK and SA using a deductive framework. The tool was pilot tested on three to four individuals to ensure its accuracy and effectiveness, and data from the pilot test were not included in the final analysis. The guide allowed flexibility to capture emerging themes during data collection. Core domains included awareness of vasectomy, perceived benefits and risks, sociocultural beliefs, family influence, and suggestions for improving acceptance. Probes were used flexibly to explore emerging issues in greater depth.

Data collection

In-depth, face-to-face interviews were conducted by APZ and PR at the rural center and by EK at the urban center. At the rural center, due to limited availability of private indoor space and high levels of crowding and noise during working hours, interviews were conducted in a relatively quieter rooftop area. Efforts were made to maintain one-to-one conversations and privacy during the interviews. At the urban center, interviews were conducted in a private setting to ensure confidentiality and comfort.

Informed consent was obtained and documented from all participants before the interviews. Participants were thoroughly informed about the study’s purpose, process, and confidentiality measures. The interviews were audio-recorded and lasted approximately 10-20 minutes. Field notes were taken during the data collection process on a writing pad. Participant recruitment and data collection continued until thematic saturation was reached, that is, until no substantially new themes were emerging from the interviews. Transcripts were not returned to participants for member checking.

Data management and analysis

Interviews were conducted in Hindi and transcribed into English using software-assisted transcription. All transcripts were subsequently manually reviewed and cross-checked against the audio recordings by bilingual members of the research team to improve accuracy and preserve meaning. A clean verbatim transcription method was used. The translated transcripts were further checked for accuracy by PK and SA. To maintain confidentiality, all files, including audio recordings, transcripts, and participant details, were securely stored in Google Drive, with access restricted to the research team. Thematic analysis was employed.

The data analysis process began with familiarization, during which transcripts were read repeatedly and cross-checked against the corresponding audio recordings. Open coding was then performed manually to identify initial codes, with coding initiated only after completing all 18 interviews. The demographic characteristics of the participants are presented in Results section. EK, APZ, and PR each transcribed six interviews, creating separate codebooks. EK, serving as the primary code editor, merged these codebooks and categorized the codes into broader themes and subthemes, ensuring alignment with the study objectives. Data saturation was confirmed when no new themes emerged. QDA Miner Lite software was used for data organization and coding. Participant quotations were incorporated to illustrate key themes, with identifiers used to maintain anonymity. All authors contributed to the interpretation of findings, ensuring transparency and consensus.

To enhance credibility, transcripts were read repeatedly, checked against audio recordings, coded independently by multiple researchers, and discussed within the research team until agreement on themes was reached. Although transcripts were not returned to participants for member checking, credibility was supported through repeated transcript review, audio cross-checking, multiple coding, and consensus-based thematic development.

Ethical considerations

The study protocol received ethical approval from the Research Advisory Committee, AIIMS, Patna (Ref. No. RD/AIIMS/Pat/2024/RAC/43). Informed consent was obtained and documented from all participants after providing them with a comprehensive understanding of the study’s objectives. Voluntary participation was encouraged, and confidentiality was maintained throughout the study.

## Results

**Figure 1:**
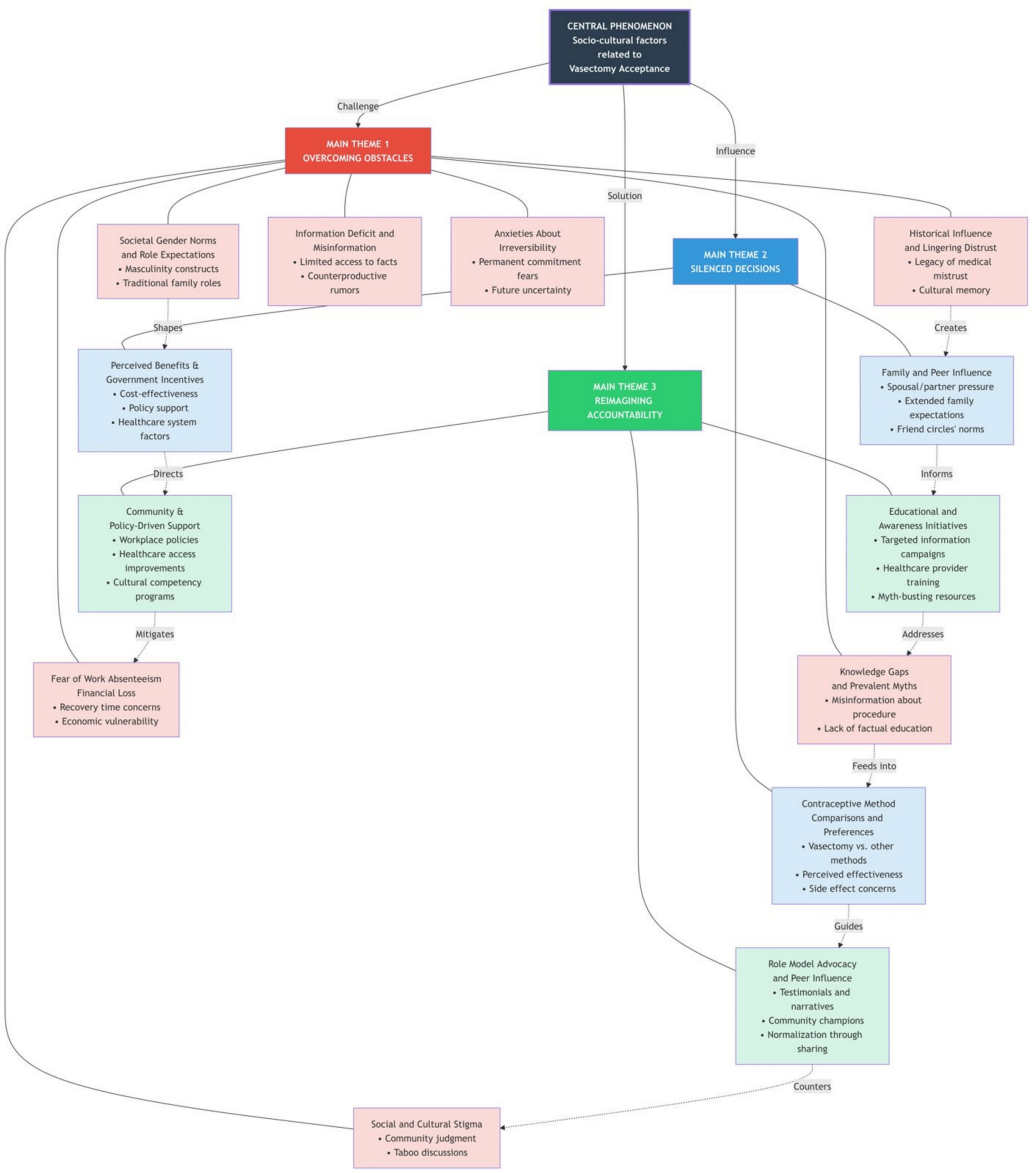
Categories and themes from the qualitative descriptive analysis

The demographic profile of participants is presented in Table 1, and the themes and categories emerging from the qualitative descriptive analysis are illustrated in Figure 1. Themes were developed from recurring patterns across participant narratives and reflected commonly expressed perceptions, barriers, and suggested solutions related to vasectomy acceptance. Four major themes, along with their corresponding subthemes, were identified from the in-depth interviews. These themes capture participants’ awareness, perceptions, barriers, and proposed strategies regarding vasectomy acceptance.

**Table 1:** Demographic characteristics of study participants (N=18) RHTC, Rural Health Training Center; UHTC, Urban Health Training Center

Participant ID	Age (years)	Occupation	Years of experience	Site of interview
P1	45	Businessman	19	UHTC
P2	63	Guard at primary health center	12	UHTC
P3	33	Driver	15	UHTC
P4	32	Engineer	11	UHTC
P5	33	Social worker	8	RHTC
P6	35	Engineer	5	RHTC
P7	40	Driver	10	RHTC
P8	60	Security guard	2	RHTC
P9	35	Security guard	10	RHTC
P10	29	Laundry shop owner	8	UHTC
P11	40	Laboratory technician	2	UHTC
P12	29	Project technical officer	5	UHTC
P13	46	Clerk	10	UHTC
P14	36	Shop owner	12	UHTC
P15	31	Teacher	5	RHTC
P16	42	Shopkeeper	15	RHTC
P17	38	Private job employee	8	UHTC
P18	44	Daily wager	30	UHTC

Theme 1: awareness, perceptions, and inherited beliefs about vasectomy

Vasectomy awareness and perceptions among study participants varied widely. Some participants considered it a valid family planning method, but several participants held misconceptions and had a limited understanding of the procedure. The existing awareness and perceptions of study participants regarding vasectomy can be further categorized under the following subthemes:

Historical Influence and Lingering Distrust

Participants, particularly middle-aged and older men, referred to the legacy of forced sterilization during the Emergency period as a source of ongoing mistrust. As one participant stated, “There was a time when vasectomy was done forcefully during Indira Gandhi's presidency for birth control” (P1, 45 years, Urban Health Training Center (UHTC)). These past events still influence men’s perceptions today, making them skeptical and unwilling to consider vasectomy.

Knowledge Gaps and Prevalent Myths

Several participants lacked a clear understanding of vasectomy and often believed it could cause impotence, weakness, infertility, or even cancer. There was a strong perception that a vasectomy leads to reduced sexual function and other health issues. One participant explained it as, “People think that after vasectomy, a man becomes weak and does not experience the same level of feelings as before” (P5, 33 years, Rural Health Training Center (RHTC)). Another participant expressed, “I fear it may lead to cancer or problems in my reproductive system” (P6, 35 years, RHTC), while a third participant shared a common concern among married men: “There is a risk of losing my sexual power” (P13, 46 years, UHTC). The myth extends beyond sexual problems, even suggesting a link to cancer despite having no scientific basis.

Societal Gender Norms and Role Expectations

A clear gender bias in family planning emerged, with male sterilization being far less accepted than female sterilization. Participants across both rural and urban settings observed that vasectomy was rarely considered, and contraception was largely viewed as a woman’s responsibility. One participant noted, “Female sterilization is normal, but only 5% of men will agree to get themselves sterilized” (P4, 32 years, UHTC). Another added, “Males don't want to undergo the procedure; instead, they prefer their female partner to do it” (P17, 38 years, UHTC). This reflects deep-rooted societal norms that discourage men from taking responsibility for contraception.

Theme 2: barriers to acceptance: fear, stigma, and practical concerns

Despite its potential benefits, multiple barriers prevent men from choosing vasectomy as a viable family planning option, which can be further understood under the following subthemes:

Fear of Work Absenteeism and Financial Loss

Concerns regarding work absenteeism and physical weakness were expressed mainly by participants engaged in wage-earning and physically demanding occupations. Several participants expressed concern about potential health issues after the procedure, including loss of strength, reduced sexual performance, and difficulty performing day-to-day activities. They noted that, while women were primarily housewives, men bore the earning responsibility, and undergoing the procedure could lead to loss of income or wages. One participant voiced this concern, stating, “If I do this, I may not be able to work properly, and I might feel weak” (P2, 63 years, UHTC). These concerns were not based on medical evidence but were rooted in cultural beliefs and misinformation circulating in the community.

Social and Cultural Stigma

In rural areas, where societal pressure and religious beliefs play a major role, there is strong cultural resistance to the acceptance of vasectomy. One participant explained, “People in my community do not believe in sterilization, especially for men” (P2, 63 years, UHTC). Another emphasized the social consequences, stating, “If a man gets a vasectomy, society will question his masculinity” (P10, 29 years, UHTC). Several participants mentioned tubectomy as a more socially acceptable option for sterilization, as family planning was perceived to be a woman’s responsibility.

Information Deficit and Misinformation

Several participants lacked basic knowledge about vasectomy, including how it is performed and its potential benefits. As one participant observed, “People do not know about vasectomy, and they also do not know how the operation is done” (P4, 32 years, UHTC). Despite the available incentives and financial support provided by the government for undergoing a vasectomy, there was still limited awareness among the study participants about these schemes.

Anxieties About Irreversibility

A common fear among the study participants was that a vasectomy could not be reversed, making them hesitant to choose it over available temporary methods. One participant expressed this concern, questioning, “If I want to become a father afterwards, what can I do? I need more information before deciding” (P15, 31 years, RHTC). These findings indicate a need for better education and counseling about the procedure, its effects, potential benefits, and reversibility.

Theme 3: family, peer, and contraceptive decision-making influences

Family and Peer Influence

Discussions with family members, friends, and doctors play a crucial role in influencing an individual's decision-making. However, in many cases, there was no discussion at all or, if discussed, spouses and family members discouraged men from opting for a vasectomy. One participant shared his personal communication with his wife, “I talked to my wife about it, but she said it was unnecessary. We will only consider it after our third child” (P13, 46 years, UHTC). This reinforces the idea that vasectomy is not a priority in family planning discussions.

Contraceptive Method Comparisons and Preferences

Participants compared vasectomy with other family planning methods; some considered it a permanent and effective solution, while others preferred temporary methods such as condoms or Copper-T because of concerns about reversibility and ease of use. One participant stated, “Male sterilization is very simple and effective compared to other methods” (P5, 33 years, RHTC). Another noted, “Vasectomy is a good solution because using Copper-T can also be dangerous” (P12, 29 years, UHTC). Conversely, a different perspective emerged when a participant expressed, “No, I think other methods are easier. There is no such difficulty” (P18, 44 years, UHTC). This highlights the need for comprehensive education on contraceptive options, ensuring men are aware of the benefits and limitations of both temporary and permanent methods.

Perceived Benefits and Government Incentives

A few participants acknowledged the benefits of vasectomy, such as reducing the burden on women and ensuring family stability. However, awareness of financial benefits and government incentives being provided was low. One participant questioned, “If the government offers money for vasectomy, why don't they advertise it properly?” (P10, 29 years, UHTC). The limited promotion of vasectomy compared to other contraceptive methods highlights a gap in public health communication strategies.

Theme 4: suggested strategies to improve acceptance

Study participants themselves suggested various strategies to improve the awareness and acceptance of vasectomy as a family planning option.

Enhanced Public Education and Awareness Initiatives

Several participants stressed the need for better educational campaigns to address myths and provide accurate information. They suggested organizing regular health camps, workshops, and village-level seminars to educate men about the procedure, along with active counseling by Accredited Social Health Activist (ASHA) workers, doctors, and other community health professionals. One participant emphasized, “People are not educated about this. The government should organize health camps” (P10, 29 years, UHTC).

Role Model Advocacy and Peer Influence

Participants also suggested using role models who had undergone the procedure and were doing well without any issues, so they could encourage more men to consider vasectomy as a family planning option. As one participant proposed, “If someone who has done a vasectomy shares their experience, it will change people's mindset” (P7, 40 years, RHTC).

Community and Policy-Driven Support

Participants also believed health workers, especially ASHA workers, could play a role in promoting vasectomy in the community. One participant suggested, “If an ASHA worker praises a man for doing a vasectomy, it will motivate others” (P7, 40 years, RHTC). There was a strong need for more focused government promotion, including house-to-house campaigns, advertisements, and community meetings. Another participant called for structured outreach, stating, “Awareness needs to be increased. Seminars at the village level should happen at least three to four times a year” (P5, 33 years, RHTC). By normalizing vasectomy and integrating it into family planning discussions, these interventions could gradually shift societal attitudes and increase uptake among men.

## Discussion

This qualitative study includes 18 in-depth interviews with married men at two peripheral health centers, one situated in a rural area and the other in an urban context in Bihar. The primary aim was to delve into the sociocultural factors influencing the acceptability of vasectomy among this demographic. The findings of this study suggest that vasectomy acceptance among married men in these settings is shaped by an interplay of misinformation, gendered responsibility for contraception, sociohistorical distrust, and anticipated social consequences. These barriers appear to operate not only at the individual level but also within family and community contexts.

A significant portion of participants had misconceptions about vasectomy, associating it with impotence, weakness, and infertility. This is in line with research conducted in various geographical regions of the world [8-11], which further highlights the widespread lack of awareness and persistence of misinformation about vasectomy. In the context of Bihar, such misconceptions appear to extend beyond limited biomedical understanding and are closely tied to local beliefs about masculinity, sexual capacity, and physical strength.

Additionally, the history of vasectomy in India still echoes in the present-day choice of men regarding vasectomy as a family planning method. Several participants, particularly middle-aged and older men, linked vasectomy to memories of India’s Emergency period, during which men were forced to undergo sterilization, causing mistrust [12]. Negative historical associations influence contraceptive choices among men today, also in India [13]. In Bihar, this sociohistorical memory appears to continue shaping perceptions, making vasectomy not merely a medical decision but also one associated with fear, mistrust, and state-linked coercion.

There was a strong gender bias in family planning decisions, where female sterilization was the favored decision over male sterilization. Even women often have to prioritize male decisions and health over their own health due to disadvantaged positions in the family and the community. Similar research findings have been seen in the study by Patel et al. and Parsekaar [14,15]. Such findings suggest that family planning remains a gender-driven issue, where male contraceptive methods are less valued, and women bear the primary burden of contraception. In Bihar, where gender norms and reproductive decision-making are often shaped within family and community hierarchies, this may further discourage men from taking responsibility for permanent contraception.

The majority of participants in our study acknowledged that they would not receive societal respect following a vasectomy and would face inquiries about their masculinity. Additional studies conducted among men in various geographical regions likewise acknowledged the absence of communal support [9]. In the present setting, this fear appears to reflect not only anticipated stigma from the wider community but also deeper anxieties around masculine identity and social standing.

Many participants in our study reported a lack of basic knowledge about vasectomy, including how the procedure is performed, its benefits, and government incentives. Similar findings were also observed in other studies [4,8,10]. While other studies conducted in other parts of India [4,7] reported that married men have enough knowledge about vasectomy, their willingness to accept the procedure is very poor. This suggests that in Bihar, as in other settings, awareness alone may not be sufficient; acceptance appears to be shaped by how information is interpreted through prevailing sociocultural beliefs and lived realities.

Our results support earlier studies showing that spousal disapproval is a significant obstacle to acceptance of vasectomy. According to qualitative research conducted in India, wives frequently worry about their husbands undergoing the procedure because they fear it will weaken them or cause marital discord (Biswas et al.) [16]. This theme is supported by international data, including a Ghanaian study, which found that wives were afraid that vasectomy would result in weakness or even divorce (Adongo et al.) [10]. In the present study, this suggests that contraceptive decision-making is relational rather than individual, and that family-level anxieties may strongly shape men’s willingness to consider vasectomy.

Many participants in this study, as well as in regional and national research, expressed a preference for temporary contraceptive methods like condoms and Copper-T due to concerns about the permanence and irreversibility of vasectomy [17]. This hesitation aligns with findings from rural Gujarat, where a descriptive study noted significant underutilization of vasectomy, with men favoring reversible options over sterilization (Puwar et al.) [18]. Similarly, a study in Ahmedabad found that fears of nonreversibility deterred men from opting for the procedure (Puwar et al.) [18]. Nationally, male sterilization rates remain below 1%, a trend attributed to insufficient promotion and persistent misconceptions about its permanence (Salve and Shekhar, 2023) [19]. These concerns are further compounded by widespread misinformation; for instance, 47% of men in rural Gujarat incorrectly believed vasectomy is irreversible, highlighting the critical need for targeted awareness initiatives such as community seminars, health camps, and couple counseling (Patel et al.) [18]. In Bihar, fear of weakness and reduced work capacity, as expressed by participants in this study, may reflect not only misinformation about the procedure but also broader cultural associations between masculinity, economic productivity, and bodily strength. This may be especially relevant in low-resource settings where male identity is closely tied to wage-earning responsibilities.

A few participants emphasized the importance of showcasing men who had successfully undergone vasectomy and were satisfied with the procedure as community role models. They believed that by sharing their positive experiences, these individuals could inspire others to consider vasectomy. This aligns with the findings of a previous study by Singh et al. [20], which demonstrated that using satisfied vasectomy recipients as peer educators significantly increased male participation in family planning. These peer educators helped dispel myths and normalize vasectomy as a safe and respectable contraceptive choice by sharing their personal success stories within the community. In the Bihar context, such peer-led communication may be particularly useful in countering stigma and building trust in ways that formal health messages alone may not achieve.

Many participants also highlighted the potential for ASHA workers and other healthcare providers to actively promote vasectomy through group and individual counseling. Supporting this approach, a 2023 Family Health International (FHI) 360 task-sharing guideline recommends training male-friendly healthcare professionals, engaging satisfied vasectomy recipients as community advocates, and providing targeted counseling sessions to address misconceptions [21]. In settings such as Bihar, where frontline health workers often serve as trusted intermediaries between communities and the health system, their role may be especially important in improving awareness, correcting misconceptions, and encouraging male participation in family planning.

Limitations

This study has several limitations. First, the sample was limited to married men aged 18 years and older, excluding younger men, single men, and women, which restricts the transferability of the findings to broader populations. Second, the study was conducted only in the field practice areas of a tertiary hospital, which may not fully represent the diverse sociocultural contexts of all regions of Bihar state. Third, the qualitative design, while providing in-depth insights, does not allow for quantitative measurement of the prevalence of attitudes or barriers. Additionally, the exclusion of women’s perspectives means the study lacks a comprehensive understanding of how spousal dynamics influence vasectomy decisions.

As with all qualitative interviews, interviewer-related influence cannot be excluded, and participants’ responses may have been shaped by the interviewer’s presence, phrasing, or perceived professional role. Although software-assisted transcription was manually reviewed by bilingual researchers, some linguistic nuances and contextual meanings may have been altered during translation from Hindi to English. In the rural setting, the interview location was selected pragmatically to reduce noise and interruption; however, the setting may still have influenced participant comfort, privacy, and openness. Because participants were recruited from those willing and comfortable discussing a sensitive topic, perspectives of more hesitant or less communicative individuals may be underrepresented. The findings are context-specific and intended to provide in-depth insight rather than statistical generalizability.

Future research could address these gaps by including larger and more diverse samples, incorporating women’s perspectives, and considering mixed-methods approaches to better understand the broader determinants of vasectomy acceptability.

## Conclusions

This qualitative study provides context-specific insight into how married men in selected rural and urban field practice settings in Bihar perceive vasectomy and the sociocultural factors that may influence its acceptability. The findings suggest that misconceptions linking vasectomy to impotence, weakness, and infertility, along with gender norms that position family planning primarily as a woman’s responsibility, may discourage consideration of vasectomy in this context. Historical mistrust associated with coercive sterilization campaigns during India’s Emergency period, as well as fears related to irreversibility, work absenteeism, and social stigma surrounding masculinity, also appear to shape men’s perceptions and decisions. Limited awareness of government incentives and inadequate promotion of vasectomy as a viable contraceptive option may further contribute to its low acceptance.

These insights may be useful for designing culturally sensitive awareness initiatives, male-inclusive counseling, and future research on male participation in family planning. Community-based education, peer advocacy by satisfied vasectomy recipients, and the involvement of frontline health workers such as ASHA workers may help address misconceptions and improve acceptability. More focused and contextually appropriate promotion of vasectomy, including communication about available incentives, may also support greater male engagement in reproductive health and help reduce the contraceptive burden currently borne by women.
